# A Device for Children’s Instrumental Creativity and Learning: An Overview of the MIROR Platform

**DOI:** 10.3389/fpsyg.2020.516478

**Published:** 2020-11-11

**Authors:** Anna Rita Addessi

**Affiliations:** Department of Education Science, University of Bologna, Bologna, Italy

**Keywords:** reflexive interaction, MIROR platform, musical creativity, flow, children’s music education

## Abstract

This article presents the pedagogical paradigm of reflexive interaction and its application in the field of technology-enhanced learning and children’s musical creativity. The main feature of reflexive interaction is the repetition-variation mechanism: something is repeated and varied during the interaction, through a continual process of imitation and variation. In the context of the MIROR project (EU-ICT Project), we exploited the educational potential of the reflexive interaction paradigm and implemented the MIROR platform, an educational device consisting of a set of softwares that implement the reflexive paradigm not only in music improvisation (as was the case for the first interactive reflexive system), but also in the field of music composition and dance. The platform was conceived as a tool to stimulate and develop musical and motor creativity in children, although it can also be used to teach a specific musical instrument. The hypothesis of the MIROR project was that reflexive interaction sustains and promotes the learning and creative expression processes of music and movement. This initial hypothesis, which stemmed mainly from the pilot study in which we observed children who were interacting with the first prototype of the interactive reflexive musical system, was subjected to a series of empirical studies conducted within the MIROR project, and was theoretically defined in order to lay down the foundations of the *reflexive interaction* paradigm and its pedagogical implications. This article summarizes the state-of-the-art of the project and brings together, in a comprehensive overview, the theoretical framework, the pedagogical concepts, the empirical studies, and the description of the MIROR platform, with the aim to reflect on the results achieved so far and point out the contribution of the reflexive perspective to the field of children’s instrumental learning and creativity.

## Introduction

Children’s musical creativity has been approached by several scholars from different perspectives and methodologies (e.g., Miell and Littleton, 2004; Custodero, 2005; Deliège and Wiggins, 2006; Barrett, 2012; Hargreaves et al., 2012; Odena, 2012; McPherson and Welch, 2018; Cardoso de Araújo, 2019). Several studies have focused on the measurement of children’s musical creativity (McPherson, 1993, 2005; Webster, 2002; Hickey and Lipscomb, 2006). In the field of technology-enhanced learning, most studies deal with internet devices, teaching strategies, composition, performance, and music therapy (e.g., Delalande, 2003; Brown, 2007; Webster, 2007; Finney and Burnard, 2009; Nijs et al., 2012; Dorfman, 2013; Bauer, 2014). According to these authors, the novelty and the educational potential of the new digital devices reside in the characteristics of interactivity and feedback in real time. However, according to De Kerckhove (1991), new technology can be considered not only as a “tool” to aid teaching, but also as providing languages and “brainframes,” also called “technology of mind,” i.e., forms of extension of the mind, just as the bicycle can be considered an extension of the body (“physical technology”), that deeply influence the processes of musical learning and the musical creativity of children.

We investigated this issue by exploring the interaction between children and the so-called interactive reflexive musical systems (from here on IRMS), a particular kind of software that responds to the user by imitating their style, like a mirror (Pachet, 2006). The first prototype of IRMS, the Continuator, was created as a tool for music improvisation for adult musicians (Pachet, 2003). We decided to study reflexive systems with children and realized a pilot study observing children interacting with the Continuator (Addessi and Pachet, 2005, 2006). On the basis of the promising results obtained in the pilot study, we decided to exploit the reflexive interaction paradigm in the field of technology-enhanced learning for fostering children’s music and movement creativity. We started the European project MIROR-Musical Interaction Relying On Reflexion (EU-ICT Programme), coordinated by the University of Bologna, with the aim to implement a new educational tool, called the MIROR platform (Addessi et al., 2013a). In the framework of the MIROR project, we proposed to extend the reflexive systems with the analysis and synthesis of multisensory expressive gesture (Camurri and Volpe, 2004), to increase their impact on the musical education of young children. In so doing, the MIROR platform was conceived as an educational device composed by several software applications exploiting the reflexive interaction paradigm not only in the field of music improvisation, as in the first prototype of IRMS, but also in the field of music composition and body creativity.

Our methodology was based on the iterative comparison between experimental observations, scientific literature and technological implementation, which allowed us to both elaborate several hypotheses, implement the platform, and develop a theoretical framework of the reflexive interaction paradigm that attempts to outline its neuroscientific and perceptual fundaments, as well as the pedagogical concepts and requirements. The softwares were implemented by applying a spiral research model (Larman and Basili, 2003), based on iterative cycles of software implementation and empirical studies with children and teachers, which involved a close collaboration between technology partners (SONY-Computer Science Laboratory and University of Genoa) and psycho-pedagogical partners (Universities of Bologna, Athens, Exeter, and Gothenburg). The results of the experiments carried out during the project produced a set of pedagogical requirements, which the technology partners used to improve the prototypes. In this way, every step of the project – from conception to development and implementation of the software – benefited from the research and skills of both the technological and psycho-pedagogical staff.

In this article, the state-of-the-art of the project will be presented. We first give a description of the IRMS and an example of observation in order to help the reader understand how the reflexive systems work with children. We then present the theoretical framework of reflexive interaction, the pedagogical concepts, the description of the MIROR platform, and an overview of the empirical studies realized with children and the MIROR applications. The article will end with a discussion and conclusion, trying to summarize the future of the project and the main contributions of the reflexive perspective to the field of children’s instrumental learning and creativity.

## The Interactive Reflexive Musical Systems

**FIGURE 1:**
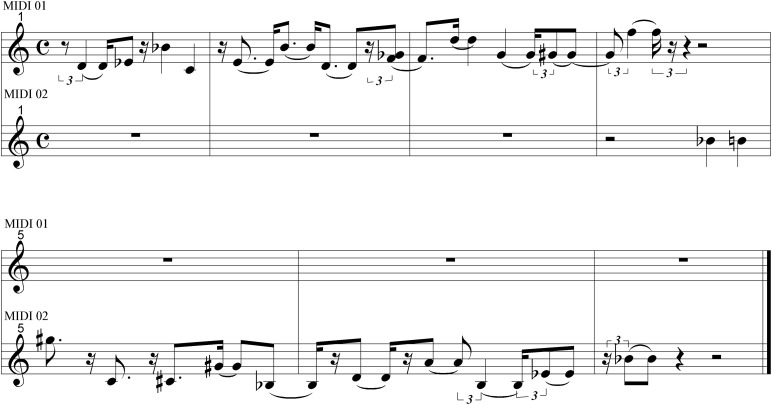
A simple melody (top stave) is continued by the Continuator in the same style (as in Addessi and Pachet, 2005, p. 23).

Reflexive interactive musical systems are a “class of interactive systems in which users can interact with virtual copies of themselves, or at least with agents that have a mimetic capacity and can evolve in an organic fashion” (Pachet, 2006, p. 360) and that identify a number of features which, although regarded as ‘non-exhaustive’ (*ibid.*), characterize such systems: the *similarity or mirroring effect*, which refers to the fact that they produce “musical sounds similar to what the user is (…) able to produce. This similarity must be easily recognizable by the user, who must experience the sensation of interacting with a copy of her/himself” (*ibid.*). In practice, the reflexive system answers by repeating the input played by the user with more or less slight melodic and rhythmic variations. The first prototype of IRMS, called the Continuator, was realized for adult musicians and music improvisation (Pachet, 2003). This system is essentially a sequence continuator: the note stream played by the musician is continuously segmented into musical phrases, which build up a model of recurring patterns. In reaction to the musical phrase played by the musician, the system immediately generates a continuation, according to the database of patterns already memorized. To illustrate the working of the Continuator, a musical example is given in Figure 1.

An important feature of IRMS is the *agnosticism*, i.e., the IRMS does not “know” the musical rules, because “no pre-programmed musical information is given to the system” (Pachet, 2006, p. 360): they “learn” automatically during the interaction with the user, by memorizing the user’s inputs. Another feature is the *scaffolding of complexity*, based on the fact that the IRMS increasingly memorizes musical material during the interaction with the user, and this “incremental learning ensures that the (IRMS) keeps evolving” (*ibid.*). Finally, the interaction is regulated by a special type of turn-taking between the system and the user, characterized by three rules: an automatic detection, by the system, of the end of the musical phrase played by the user; the system generates the phrases which have the same length as the last phrase played by the user; priority is always given to the user, meaning that if the user starts playing while the system is still playing, the system stops and restarts from step 1 (Pachet, 2003, 2004). Descriptions related to higher levels of IRMS structure are not discussed here, as they are not relevant for our purpose (see Pachet, 2003, 2006; Pachet et al., 2011, for more details).

## An Example of a Child’s Interaction in a Reflexive Environment

The exploratory experiments we realized with children and the first prototype of IRMS, the Continuator, immediately showed the efficacy of these kind of systems for enhancing children’s creative musical experiences (Addessi and Pachet, 2005, 2006). It was possible to observe that children conversed with the system by exploring the keyboard, inventing sounds and musical phrases, and original ways of producing sound. They listened attentively to their own productions and to the system’s responses, shared the rule of turn-taking and invented new rules, co-built together with the system and with companions, creating musical improvisations full of expressivity and participation. It was observed that the children’s attention span was higher when they played the keyboard with the Continuator than without it. The IRMS, with their mirror behavior, seem to generate very complex reactions in which children develop a set of constructs on their own self and on the “other,” thus supporting the child in building a “musical self,” that is the child’s musical style and identity. What caught our attention in the exploratory studies was that the interaction with these systems showed some similarity with that interaction among humans, in particular the mechanism of repetition and variation, and of turn-taking that was observed in infant-adult interaction (Stern, 2004; Imberty, 2005; Papoušek, 2007).

To understand in detail how reflexive interaction works, we describe a short session of an 8-year-old girl playing a keyboard connected to MIROR-Impro, one of the applications of the MIROR platform, which is an augmented version of the Continuator, the first prototype of IRMS:

“*The little girl plays two consecutive notes, C2 and A2, and then stops to wait for the response of the system. The system responds by repeating the same notes. The child then plays a single note, G2, and the system responds with a single note but this time introduces a variation: she plays C3, thus introducing a higher register. The girl, following the change introduced by the system, moves toward the higher register and plays a variant of the initial pattern, namely: D2-A2-E2-C3, and introduces a particular rhythm pattern. This “re?exive” event marks the beginning of a dialog based on repetition and variation: the rhythmic-melodic pattern will be repeated and varied by both the system and the child in consecutive exchanges, until acquiring the form of a complete musical phrase. At some point in the dialog, the child begins to accompany the system’s response with arm movements synchronized with the rhythmic-melodic patterns, creating a kind of music-motor composition”* (Addessi et al., 2017b, p. 4).

This observation show us that the girl moves from the random playing of two notes to an elaborate succession of rhythmic-melodic patterns, also interpreted through body movements, in particular of her arms. In this example you can observe the fundamental features of reflexive interaction: the presence of turn taking; the system response lasts as long as the last phrase played by the girl, this way leading to a regular timing of turns; the little girl’s attention increases when the system imitates the phrase she played; the dialog is constructed not only by the system, nor only by the girl, but is “co-constructed” by the girl together with the system; the presence of co-regulation, i.e., each partner regulates her/his behavior based on the behavior of the other partner (Fogel, 2000); the two partners are both able to imitate each other; finally, the girl realizes she is being imitated.

## The Theoretical Framework of Reflexive Interaction

Starting from the exploratory study realized with children, several theories have been taken into account to explain human behavior when interacting with reflexive systems, and therefore outline a reflexive interaction theoretical frame with pedagogical implications. This section presents a summary of the theoretical framework published in Addessi (2014) and introduces some new contents recently elaborated.

In the field of human-machine interaction studies, we can find traces of the paradigm of reflexive interaction in Turkle (1984), who discussed how digital technologies represent a sort of “Second Self” of the users, because they would deeply affect the psychological and social life of human beings and the development of their identity. From this point of view, the IRMS can be interpreted as a “musical” Second Self of the users. However, we can argue that the theme of the “mirror” and the “sound mirror” has characterized Western culture for a long time. References range from Ovid’s myth of Echo and Narcissus (43 BC-18 A.D.; *Metamorphoseon libri* XV), to the *in eco* procedures of Renaissance and Baroque music. In the *Teoria degli Affetti* (Vincenzo Galilei, *Dialogo della musica antica et della moderna*, Florence, 1581) and in the *Affektenlehre* (Athanasius Kircher, *Musurgia Universalis*, Rome, 1650) we find another form of the “reflexive” power of music, that is, to represent human affections through sounds, in an empathetic way.

In the following sections we will present the theoretical framework of the reflexive interaction paradigm. These sections will also refer to our experiments because we elaborated the theoretical framework during the work on the basis of an iterative comparison between our empirical observations and the scientific literature. In fact, in order to explain the processes observed during the empirical studies with children in reflexive environments, we referred to several models and theories from different fields of research: psychology, neuroscience, pedagogy, music analysis. However, the reflexive interaction paradigm does not coincide with any of these theories and represents an original approach to children’s learning and creativity.

**FIGURE 2:**
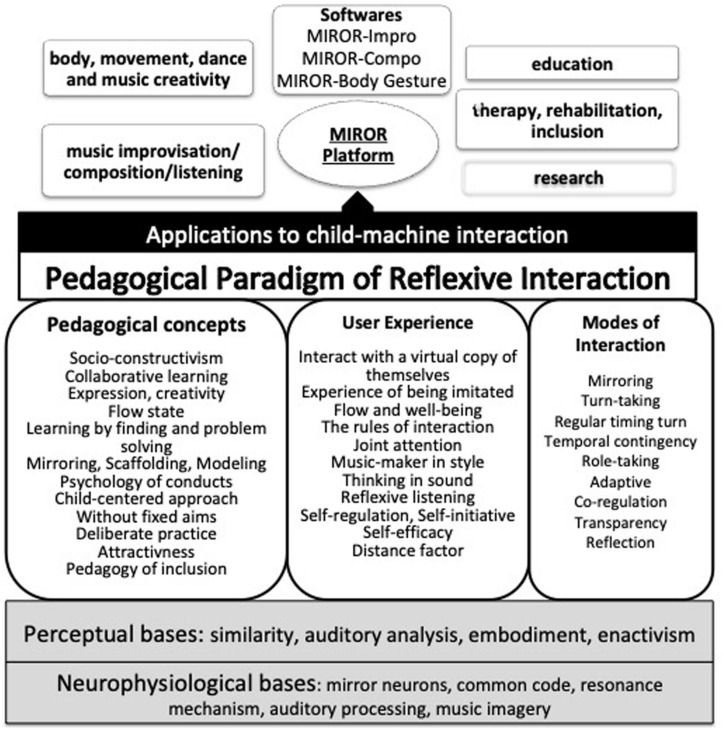
Overview of the theoretical framework of the pedagogical paradigm of reflexive interaction.

An overview of the theoretical framework of the reflexive interaction paradigm is shown in Figure 2.

### Repetition and Variation, Turn-Taking, Co-regulation

The main feature of reflexive systems and reflexive interaction is the repetition-variation mechanism: something is repeated and varied during the interaction, through a continual process of imitation and variation. Turn-taking and co-regulation between the partners are also fundamental. The turn-taking allows the child to produce, to feel listened to, and to listen. During the reflexive dialog, the child and the system adapt to each other and co-regulate the content, the rhythm, and the shape of the interaction. The concept of co-regulation refers to Fogel (2000), which described a series of ways in which two partners regulate their behavior reciprocally.

The mechanism of repetition-variation is also the basis for some schools of musical analysis of a semiotic nature (Ruwet, 1966) and for the theories on the perception of similarities in music listening (Deliège, 2001; Toiviainen, 2007). Several studies indicate that the mechanism of repetition-variation plays an important role in the development of infant musicality and represents one of the ontological foundations of human musicality (e.g., Dissanayake, 2000; Imberty, 2005; Mithen, 2005; Malloch and Trevarthen, 2008). In the first months of life the processes of imitation, self-imitation, recognition, and repetition-variation develop and structure the child’s self and her/his interaction with humans and the environment (e.g., Papoušek, 1996; Nadel and Butterworth, 1999; Imberty, 2005; Gratier and Apter-Danon, 2008; Addessi, 2020). Anzieu (1996) defines this type of childhood experience as the “musical wrapping of the Self,” which is expressed by one of the most archaic forms of repetition, the echo, and that represents the first embryo of the personality perceived as a unity. The reflexive interaction is characterized by a dynamic process which also involves affective and emotional conditions, which Stern (2004) calls “affective contours.”

In recent decades, the repetition-variation mechanism has also found interesting interpretations in light of recent studies in neuroscience. Zatorre (2012), for example, highlighted some neural and cognitive mechanisms which allow the transformation and manipulation of pre-existing music mental representations. Hellmuth Margulis (2014) pointed out the neurobiological aspects of repetition in music, taking into consideration the complexity and multiplicity of its manifestations. The ability to replicate the behavior of others can find its neuroscientific foundations in the mechanism of the mirror neuron system, that is a network of neurons which become active during the observation and/or execution of actions (Rizzolatti et al., 2002). These researchers speculate that there is an evolutionary mechanism, which they called *resonance*, through which the visual descriptions of motor behaviors are matched directly with motor representations of the observer who is observing those same behaviors. Further studies have shown that the resonance mechanism also works through the auditory channel (see Kohler et al., 2002). As Leman (2007) points out, there is evidence that “mirror neurons are amodal, in the sense that they can encode the mirroring of multiple sensory channels” (p. 91). Therefore, the interaction in a reflexive environment would stimulate a *resonance* mechanism in the motor areas of the child’s brain and can be interpreted by means of the *enactive approach*, which sees the interaction between mind, body action, and environment as the fundament of the mental processes (Varela et al., 1993). From this perspective, reflexive interaction through the ear canal, as happens during the interaction with MIROR-Impro, would stimulate a *resonance* mechanism in the motor areas of the child’s brain.

### Pedagogical Concepts

The paradigm of reflexive interaction can be referred, in the first instance, to the socio-constructivist perspective, where attention shifted from individual to collective and collaborative processes (see Miell and Littleton, 2004; Burnard, 2007, 2012; Cross et al., 2012; Schiavio and Høffding, 2015); creativity is considered as a means of expression for the child and, consequently, one of the first objectives of music education (Delalande, 1993; Baroni, 1997). In agreement with Baroni, “we believe that we can justify a principled stand: there is an absolute necessity that the time of expression takes precedence over the time of learning. It is not just because the construction of expressive objects is our main goal, but also because this process is the only effective and convincing motivation for learning” (1997, p. 141).

The pedagogical potential of the reflexive interaction lies in the cognitive conflict stimulated by the repetitions and variations during the dialog with the system, which give rise to problem-finding and problem-solving processes that are the basis of learning. In our studies, we observed behaviors of the exploratory type characterized by the discovery of both the new “partner” and multiple musical ideas. We also observed creative behaviors, in which the child focuses on some ideas and elaborates them during the dialog with the system. It was possible to observe personalized cognitive and musical styles because the way in which children play during the interaction reveals their stylistic competence as music-makers (Addessi and Pachet, 2005, 2006).

In reflexive interaction, the focus should be not on the final product but rather on the child and the interactive process. An important characteristic of the IRMS, in fact, is that they are not programmed with fixed objectives, like, for example, the ear training softwares. The child is not asked to achieve a goal previously set by the machine, such as tuning an interval, producing a rhythm, etc., and the system does not “judge” the child’s production. In a normal session with IRMS, the first musical input, from which all subsequent musical ideas will be generated, comes from the child, and the system immediately adapts itself to the child’s input. The interaction continues with reciprocal imitation and variation, and the objectives develop during the interaction itself. The final product is therefore not predetermined by the machine. That does not mean it is not possible to make a list of music and motor skills that children can acquire when interacting with IRMS, but these skills should be a by-product of the interaction and not goals set *a priori* by the system. This kind of approach develops autonomy, intrinsic motivation, prolonged attention spans, self-regulation, self-learning, and self-initiative.

This also explains why the attractiveness of the reflexive interaction is not based on extrinsic motivations in the form of rewards or of colored interfaces “for children,” but on the child’s involvement in a scaffolding of complexity which avoids the experience of monotony and mere repetition. In fact, it was observed that the children’s attention span was increased when they played the keyboard with the Continuator (Addessi and Pachet, 2005).

In Addessi (2014) we initially suggested that IRMS exploit the concept of “zone of proximal development” (Vygotsky, 1962), by establishing a learning interaction between children and the system itself. In fact, this is true to the extent that learning, in the interactionist approach, develops thanks to the interaction between two subjects, in our case between children and the machine. The system plays a role of “scaffolding” and “modeling” (Vygotsky, 1962; Bruner, 1983), in the sense that it supports the child during improvisation and composition, providing him/her, by means of its mirror response, turn-taking and regular timing of turns, a discursive structure that allows the child to invent, develop and carry out new musical ideas. For this reason, reflexive interaction produces a child-centered approach. Nevertheless, the reflexive interaction paradigm cannot be fully interpreted with the Vigotskyian model. In fact, such systems do not behave like the adult who, in Vygotsky’s concept, plays the role of the most competent partner and thus the tutor. IRMS do not have a higher musical competence than that possessed by the children; as a matter of fact, they learn from the children themselves, while interacting with them. Therefore, the interaction that takes place between the child and an IRMS is also close to the model of interaction between peers, as described, for example, in the concept of “collaborative learning” by Dillenbourg (1999): a “*situation* in which *two or more* people *learn* or attempt to learn something *together*” (p. 1). The IRMS therefore perform a dual role, as a virtual tutor and partner, which enhances children’s musical creativity as well as their socialization.

The ability of IRMS to imitate the style of a human playing the keyboard, and to maintain children’s attention for long periods of time, has also been interpreted through the theory of flow introduced by Csikzsentmihalyi (1996). The theory of flow is closely related to the concept of creativity and describes the psychological state of “optimal experience” that results from a balance, perceived by the subject, between challenges and skills. In short, IRMS were defined as “flow machines” (Pachet, 2006). This hypothesis has been partially supported by empirical studies carried out with children, which showed that during the interaction with the Continuator and the MIROR-Impro, children reach higher levels of flow than without the system (Addessi et al., 2006, 2015a).

The MIROR platform can been defined as a “device” to enhance and motivate children’s musical and motor creativity (Addessi, 2015); in other words as a tool to support the musical “conducts” of children. The word “conduct” refers to the *psychologie des conduites*, introduced by Pierre Janet, Jean Cleparède, and Jean Piaget (Galimberti, 1992). The musical conducts are defined as a set of actions coordinated by the purpose of making music (Delalande, 1993). As Frapat (1997) points out, in this educational perspective, the concept of “device” takes on a fundamental role since it represents a “concrete mediation” that the teacher should identify and use in every specific situation, with the aim to allow children to concentrate their attention on the sound and movements.

An important educational aspect of reflexive interaction is that it enhances the listening to one’s own productions and those of the system and companions. In fact, the main channel of interaction between children and the machine is listening: this encourages children to think “in sounds,” which is considered the first step toward playing by ear and from memory (McPherson, 2005). It also encourages children to listen to their own sound productions, of fundamental importance for developing explorative and inventive musical conducts (Delalande, 1993). The listening conducts of children during the interaction with MIROR-Impro, for example, are particularly rich and varied: surprise, analytical concentration, symbolic, aesthetic, empathetic, collaborative, bodily, autotelic, multimodal. In particular, the dialog with a MIROR application generates a type of “intertextual” listening, during which the children are called to interactively build and reconstruct the fragments of their musical discourse.

Studies carried out so far have also shown that reflexive interaction can be a strategy for the teacher to stimulate the communicative behavior in situations of disability and inclusion. The “optimal experience” (Csikzsentmihalyi, 1996) generated by the interaction with reflexive systems creates states of “well-being” and “optimal experience,” demonstrating a strong therapeutic potential (cf. Anagnostopoulou et al., 2012; Ferrari and Addessi, 2016, 2019).

### Pedagogical Requirements of Reflexive Systems

A list of pedagogical requirements of the IRMS has been created which focuses on the modes of interaction and children’s experience. These requirements merged from the technical characteristics of the IRMS (Pachet, 2003, 2006), from the observations made during the pilot study (Addessi and Pachet, 2005, 2006) and the implementation of the MIROR systems (e.g., Addessi et al., 2015a, 2017a), and are strictly linked to the theoretical framework and pedagogical concepts of the reflexive interaction paradigm. A synthesis of the pedagogical requirements is shown in Figure 2.

#### Modes of Interaction

The modes of interaction between IRMS and children, in order to be called “reflexive,” should have the following characteristics:

• *Mirroring*: this is based on the mechanism of repetition – variation, which is the core itself of reflexive interaction.
• *Turn-taking*: this allows the child to produce, to feel listened to, and to listen. The rule of turn taking is readily learned by children and is applied in an intuitive manner, without the need for an explanation.
• *Regular timing of turns*: the phrase generated by the system should have the same length as the last phrase played by the child, in order to make the dialog as natural as possible. In fact, in infant-adult interaction the partners spontaneously reach a sort of “pulse” based on the regular timing of turns (Stern, 2004; Malloch and Trevarthen, 2008). In our experiments, we also observed that when the answer of the system is longer than the input played by the child, the child dislikes the behavior of machine (Addessi and Pachet, 2005).
• *Temporal contingency*: the system’s response must respect a time-lapse interval that allows the child to perceive the response in a causal relationship with the musical phrase, which she/he played. In MIROR-Impro the temporal threshold that seems to work best is between 400 and 600 ms, but other interval time ranges can be set.
• *Role-taking*: the interaction with an IRMS is based on role-taking since any intervention of each partner has something of the phrase played by the other partner.
• *Adaptive*: the system constantly adapts to the user’s musical and motor style, i.e., the style (musical and learning) of each child.
• *Co-regulation* (Fogel, 2000): by means of a reciprocal imitation and variation of the musical inputs, the child and the reflexive system co-regulate their dialog by adapting their behavior to each other.
• The system should have the properties of *transparency* (i.e., children, in fact, interact with IRMS only or mainly by playing music or through movement), and *reflection* (Folkestad et al., 1998), because the system itself guides the user to understand the rules of interaction.

#### User Experience

The user experience should be characterized by the following phenomena:

• *Experience of interacting with and manipulating a virtual copy of themselves*: children must feel they are interacting with something similar to themselves. Imitation, self-imitation, imitation recognition. Perception and production of variations.
• *Experience of being imitated*: reflexive learning is not learning by imitation, but rather is activated by the feeling of being imitated.
• *Flow experience* (Csikzsentmihalyi, 1996): during the reflexive interaction children should reach a high level of *flow* and well-being.
• *The rules of the interaction*: children should learn the rules during the interaction itself (turn-taking, repetition, repeats with variations, or “errors,” etc.). Children should learn to respect the rules, to enforce them and to invent new ones.
• *Joint attention*, *in pairs or group sessions*: children play, listen and explore together, observing their partner’s reactions and the system’s reactions together with their partner.
• *Music-maker in style*: in children’s musical exploration and improvisations, we should observe the presence of personal styles, in the way they produce the sounds, their way of handling the instrument. The IRMS, with their mirror-like behavior, are able to enhance the child’s individual style.
• *Reflexive listening*: the IRMS develop an “intertextual” way of listening where the children are involved in dialoguing with the fragments of their own sound language re-launched by the system.
• The *distance factor* (Bertolini and Dallari, 2003): the children should be able to stop playing whenever they want, thus preserving the “distance” with the machine.

#### The Requirements for Reflexive Embodied Interaction

With the MIROR project we attempted to implement reflexive interaction also in the field of embodied and multimodal technology. The idea was to create a system able to capture the movements of the children and transpose them into sound (Addessi et al., 2013b). Embodied reflexivity concerns the connection between the movements produced by the children and the sounds produced by the system, which should “reflect” the qualities of the children’s movements, in order to make them perceive that the system’s sounds represent a sound copy of their movements. Thus, the reflexive requirements were described for the embodied reflexive systems as follows:

*Mirroring*: during the interaction with the system, the user should have the perception that the sounds produced by the system are a “copy” of her/his movement; e.g., tied/detached, long/short movements; tied/detached, long/short sounds, etc.

*Repetition and variation*: the system should introduce several sound variations in real time, creating a scaffolding of complexity throughout the interaction.

*Turn-taking*: during the interaction the child should have the possibility to both alternate her/his movements with the sounds produced by the system and to listen to the sounds while they are moving. Therefore, we introduced two kinds of interaction: turn-taking and simultaneity.

*Regular timing of the turns*: in the case of turn-taking, the system’s reply should have the same duration as the child’s input.

*Adaptive*: the child should not be asked to adapt her/his movements to the system, on the contrary, the system should “learn” from the way each child moves her/his body, and adapt the sounds to the qualities of her/his movements.

## The Miror Platform

The MIROR platform was conceived as an advanced cognitive tutor composed by several software applications, which exploit the reflexive interaction paradigm in the field of technology-enhanced learning for children. The platform was not designed for teaching a specific instrument, though it can also be used with this objective. It has been conceived rather as a device to stimulate and enhance children’s musical and movement creativity.

### Target Groups

The MIROR platform was conceived for boys and girls from 2 to 10 years old. It was designed for children in nursery schools, kindergarten, primary schools, music schools, dance schools, children’s recreation centers, children’s hospital departments, and social inclusion contexts, such as immigration and community centers. The platform can also be used in therapeutic and rehabilitation settings. Another target group are the teachers themselves: the platform can in fact be also used to develop teachers’ music and motor creativity. Finally, the children can use the MIROR applications at home, together with their parents, brothers, sisters and friends.

### MIROR Applications

Three applications have been implemented during the MIROR project: the MIROR-Impro, an augmented version of the Continuator, dedicated to music improvisation; the MIROR-Compo, a software that uses reflexive interaction to allow children to compose music, and the MIROR-Body Gesture, which supports children’s music and body movement invention. Below a brief description of the three applications implemented during the MIROR project is given.

#### MIROR-Impro

**FIGURE 3:**
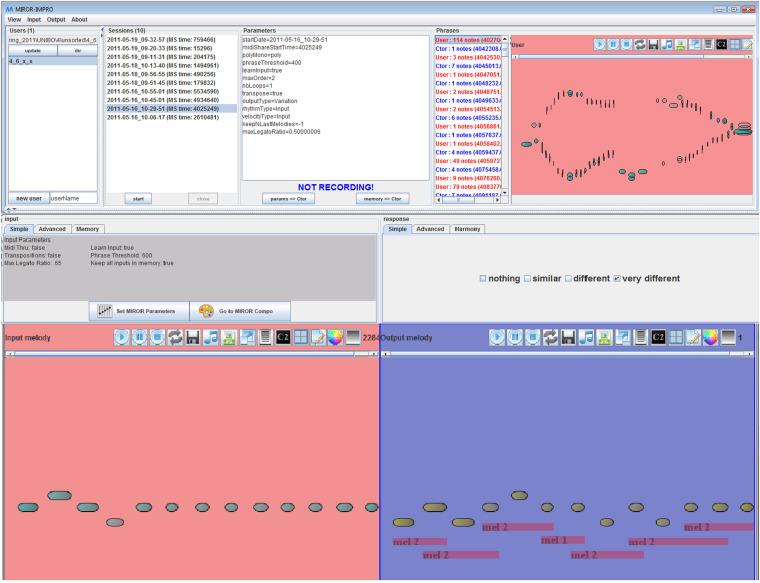
Example of MIROR-Impro interface (version 3.10). In the two lower panels you can see the user’s input (on the **left**) and the response generated by the system (on the **right**). The upper panels contain, from left to right, the list of users, the numerical display of the inputs and outputs, the parameters, the turns of the system and user, the panel for the user to program the session and the type of response of the system. In the right middle panel, you can select whether the system responds or not and, if it does, the different types of reflexivity: *nothing* = no system response; *similar* = the system’s response contains many repetitions of the user’s input; *different* = the response of the system contains few repetitions; *very different* = the system’s response is very different compared to the user’s input.

The application MIROR-Impro can be used by children of every age, and must be connected to a MIDI keyboard or another instrument, for example the drums. In a normal session, when the children play something and then stop, the software answers with a “reflexive” reply based on the input played by the child. In other words, the system’s reply repeats and varies the user’s input, producing a sort of sound mirror of what the child played. The child/ren can improvise and dialog with the system as a sort of partner, trying to discover what is the same or what changes. The reflexive interaction develops a dialog between the child and the system, during which the improvisation process takes shape and develops. The main goals of this application are to help children to explore the sounds and the instruments, to dialog and interact by means of the sounds (mirroring, turn-taking, and co-regulation), to improvise with sounds, and to educate and strengthen their auditory and creative skills. MIROR-Impro preserves the characteristic of the Continuator, but it has new features such as: (1) New kinds of system response, according to the degree of repetition-variation and based on the segmentation of the original input (similar, different, and very different); (2) An interface in which the user can more easily modify the set up and see the graphical representation of her/his input and system output; (3) The possibility of transcribing the child’s and the system’s musical phrases in traditional music notation; (4) The tool to save the musical phrases (both of the child and the system) in various formats, and to share and archive them (For more technical details see Pachet et al., 2011; Addessi et al., 2013a). Figure 3 shows an example of the interface of MIROR-Impro during a session.

#### MIROR-Compo

**FIGURE 4:**
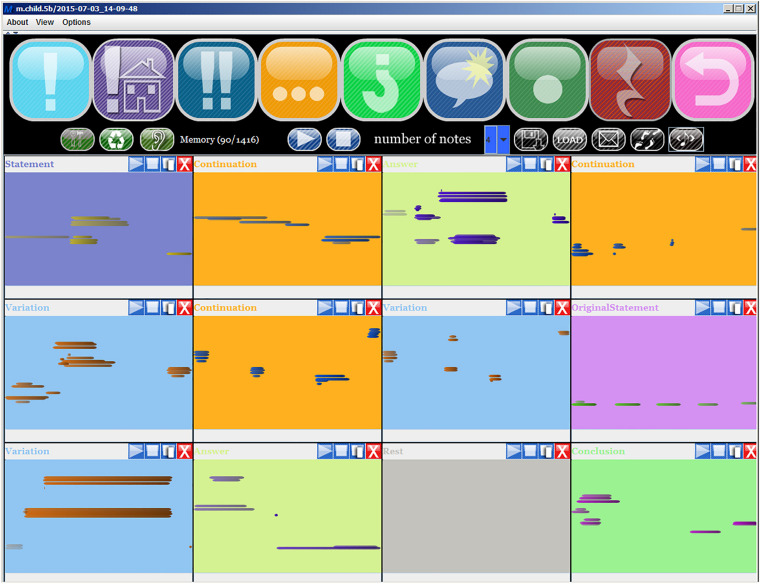
Example of MIROR-Compo interface. The upper icons indicate the type of sentence that the user may ask the system for, by clicking on them, and are, in order of presentation: *Create Statement*, *Original Statement*, *Repeat the last chunk*, *Create Continuation*, *Create an Answer*, *Create a Variation*, *Create a Conclusion*, *Rest*, *Backtrack*. The rectangles below display the initial sentence indicated by the child to start the composition (*Original Statement*) and the subsequent phrases the child chose from among those generated by the system: *Continuation, Answer, Continuation, Variation, Continuation, Variation, Original Statement, Variation, Answer, Rest, Conclusion*.

The application MIROR-Compo supports children in composing music: it is a kind of “musical scaffolding,” which helps children to create musical form with their style and musical taste. It is most suitable for 6 to 10-year-old children. The session begins with an improvisation with MIROR-Impro. The child can choose one of the musical phrases created during the session with the MIROR-Impro, and use it as the opening sentence of the composition, inserting it in the MIROR-Compo. At this point the system will provide the child the opportunity to choose from among different “actions,” i.e., a new musical phrase that functions as a *Continuation* of the initial sentence, or a *Variation*, or a phrase in the form of *Answer*, or *Conclusion*, and so on. The system generates the sentences and the child can choose whether to accept or reject them. She/he can play back the music and eventually end it when she/he thinks the composition is complete. The educational potential of this application lies in guiding children in the creation of music, in symbolic musical storytelling, and in collaborative compositions. Reflexivity is present in the sense that MIROR-Compo produces musical phrases “re-flexing” the initial sentence produced by the child during the improvisation with MIROR-Impro. Thus, the practices with MIROR-Compo offer the interesting opportunity to promote mutual reference between improvisation and composition (For technical details see Pachet et al., 2011; Addessi et al., 2013a). Figure 4 shows an example of the interface of MIROR-Compo during a session.

#### MIROR-Body Gesture

**FIGURE 5:**
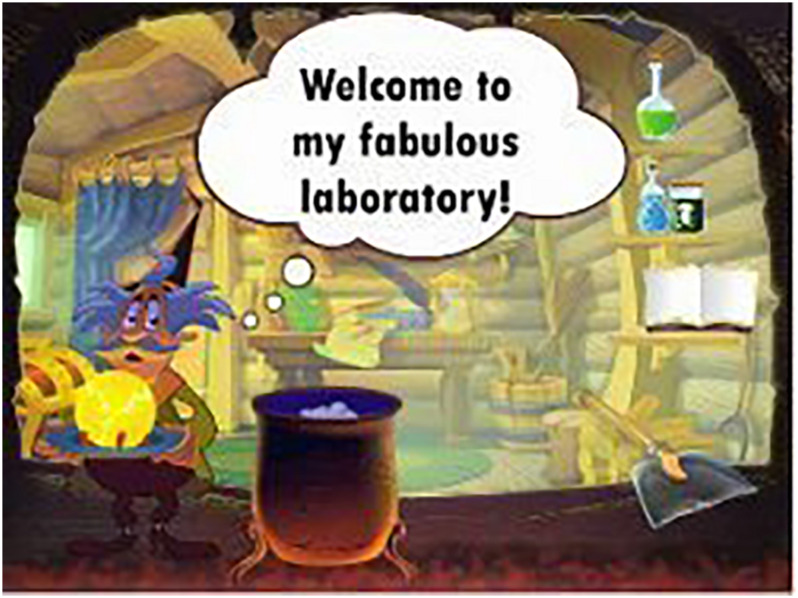
Image of the beginning of the story proposed by MIROR-Body Gesture: a wizard will guide children to explore different characters and their movements.

The MIROR-Body Gesture was originally conceived as a system able to capture the qualities of the children’s movements and translate them into “reflexive” sounds, i.e., which have the same qualities as the movement produced by the children (fast/slow, heavy/light, etc.). The children should be able to create music by moving their own body inside a sensorized space and to interact with the sounds produced by their own movements. In this way, the MIROR-Body Gesture should support children in exploring the musical qualities of their own body and movements, and, on the other hand, the embodied qualities of the sound and music. The MIROR-Body Gesture application exploits and amplifies a number of elements of gesture analysis proposed by Rudolf Laban (1879–1958): we aimed to take advantage of this theoretical and practical framework coming from the context of dance education, in order to develop children’s motor and musical skills through reflexive technologies. The MIROR-Body Gesture implemented in the framework of the MIROR project does not use sensors but the kinect, and focuses mainly on the exploration of rhythm, melody, and harmony (for technical details see Volpe et al., 2012; Addessi et al., 2013a; Varni et al., 2013). An example of the interface is shown in Figure 5. The original idea of MIROR-Body Gesture is still ongoing and not fully implemented.

### The Practices and the Teacher’s Role

We can distinguish three types of practices that can be implemented with the MIROR platform applications: *Practice 1*: the children interact with one or more applications of the MIROR platform; *Practice 2*: the children use one or more applications of the MIROR platform together with the teacher; *Practice 3*: the MIROR platform is used by the teachers, for example, but not only, for the training of teachers.

#### The Teacher’s Role

The role of the teacher, and the adult in general, may change according to the MIROR application, the age of the pupils, the contexts (for example: compulsory school, music school, and home), and the educational perspective and curriculum.

Generally speaking, the adult’s role should not be that of “explaining” to the child what to do during the interaction, because by their nature the MIROR systems should be “adaptive,” “intuitive” and “transparent,” i.e., they should allow the child to learn the modes of interaction with the system while interacting with it. This is particularly true with MIROR-Impro, because the children interact with the system by playing a keyboard or another instrument. With MIROR-Impro, the child can interact with the system by her/himself, so it is always desirable and appropriate to provide a period of individual exploration, without mediators or the intervention of the adult, unless requested by the child. With MIROR-Compo and MIROR-Body Gesture, depending on the age of the children, the adult could be necessary to support the child/ren in using the interface of the system.

For all three MIROR applications, we suggest that the role of the adult/teacher should be that of a mediator, scaffolding and modeling, adopting the following methodological approach (for the teacher, but also a parent or therapist, etc.).

*Setting preparation*: in *Practice 1* and *2*, the adult’s role may be to prepare the environment, organize the equipment, and make the system work. In order to do this, the adult must plan whether the child will play alone, in a group with other children, or with the adult’s mediation/guidance. Depending on the age of the children, with MIROR-Compo it could be necessary for the adult/teacher to also support the children in understanding how to start and use the software.

*Observation*: the teacher observes the children’s interaction with the system and their musical and motor “conducts.” The observation phase is needed especially at the beginning of the activities, because, as the experiences so far gathered with the children have shown us, it is important that – before carrying out the planned activities – the adult carefully observes the type of interaction that develops between the child and the system. Therefore, it is important that the adult thinks and plans in advance with which modalities she/he will intervene, to “re-launch” the music ideas born from the children/system interaction, in a way that is motivating for the children themselves. In the observation phase, it could be helpful to use a check-list or an observational grid, to register, for example, the musical conducts of children or the flow state (see the grids in Addessi et al., 2015a, 2017a).

*Re-launch*: in this phase, the adult interacts with the children and becomes an active participant in the learning context (Frapat, 1997). The teacher sets the conditions necessary to accomplish the transition from the children’s spontaneous exploration to the intentional invention. According to the *psychology of conducts* (Jean Claparède, Pierre Janet, and Jean Piaget), the *re-launch* concerns the “motivation” and not the “execution” (Delalande, 1993). This means that the adult does not tell the child what to do, but offers her/him a *motivation* to do something, stimulating the intrinsic motivation. The teacher can use the MIROR applications as a device to motivate children to focus on the sound and movements, to promote and enhance the children’s creativity, to implement the musical curriculum by assigning tasks to the pupil, and/or suggest several practices for deliberate practice. To achieve this stage, the adult/teacher/educator can use the following strategies (Vygotsky, 1962; Bruner, 1983): *mirroring*, that is imitating what the child does, thus strengthening her/his musical behaviors; *scaffolding*, that is building a structure that allows the child to use the system in the most suitable way for her/him, respecting the child’s cognitive and musical style; *modeling*, that is supporting the child’s exploration/invention by helping her/him carry out and complete the activities she/he started.

*Reflection, evaluation, and metacognition*: the reflection on the interactive experience and the “outcome” (i.e., the recording of the music improvisation session with the MIROR-Impro, the music composition realized with MIROR-Compo, the dance composition with the MIROR-Body Gesture) can be introduced and scaffolded by the teacher, depending on the age of the children, by means of a discussion among peers and the peer evaluation of the “outcome.” Teacher can suggest and guide the children to elaborate and share several criteria for evaluating their outcomes. Reflection can be also realized by means of other activities, for example by inviting the children to draw their experience with the system, to represent their dialog with the MIROR-Impro using traditional and/or spontaneous music writing, to improvise some dance movements by listening to their composition or performance, etc. The reflection and evaluation of the outcome led by the teacher can represent a moment of metacognition, which allows children to share their experience with their companions, make a synthesis and conceptualize their musical experience with the system. Reflection and evaluation can be a device to re-launch a new path of explorations, performance, composition with the platform.

It is important to stress that reflexive systems, by their nature, introduce reflection on the outcome during the interaction itself. In fact, the reflexive mechanism is based on continuous reflection about what is similar and what is different between the input and the system’s answer. This kind of reflection is a very fast process that the children carry out during the interaction with the system, without any adult intervention. Some children, for example, love to produce long musical phrases and then listen to the system’s answers. During collaborative sessions, the children also share their reflections and thoughts about their productions, the system’s answer, how to interact with it, and to invent new ways to play or compose. This musical dialog, which every child can arrange according to their own style, can be destroyed by an adult’s verbal, conceptual or reflective intervention during the interaction itself. Wallerstedt and Lagerlof (2011) affirm that in some cases the children are not able to start the interaction with MIROR-Impro without the help of an adult. However, they did not demonstrate this. On the contrary, the empirical observations described in their article show that the children get annoyed or dislike the system only when the adult is present and invites the children to reflect during the session. In fact, it needs to be taken into account that each child has her/his own time to start the dialog and discover the rules of interaction with the system (turn-taking, repetition-variation, etc.), and the adult should be careful not to force the time and wishes of the child/ren.

### Some Scenarios in Nursery, Kindergarten, Primary School, Music School, Dance School, and at Home

In this section we will introduce some scenarios with the MIROR platform in different contexts. The MIROR applications are flexible and suitable for different scenarios and ages. The fact that they are adaptive makes these applications potentially suitable for all ages and contexts, with specific adaptations. As we said, the goals are not fixed by the system and several, different objectives can be planned by the child/ren and/or the teacher on the basis of the context, activity, scenario.

In childcare services, for example, reflexive interactive systems are particularly suitable for supporting exploration activities, in this case sound and motor, which are fundamental in the child’s growth at this age. We suggest therefore to focus more on sound and bodily exploration in early childhood, on exploration and musical invention based on symbolic play (let’s pretend, story-telling) in the kindergarten, and on exploration, invention and composition activities in the primary school.

In music schools, the teachers can use the system as a support in formal instrumental education. MIROR-Impro can be useful to teach music improvisation skills, based on collaborative playing and musical dialog. The music teacher can use MIROR-Impro and MIROR-Compo to work on certain focuses, such as a rhythmic-melodic pattern, collaborative playing, and composition. These applications are suitable for “deliberate practice” (Ericsson, 1997) at home as well as in the music-school and can be used in alternation with the teacher’s lesson, supporting the musical curriculum.

These systems can become the children’s sound companions, can be placed in a corner, at school and at home, and be available to children for extemporaneous explorations in individual sessions or with their friends, or brothers/sisters, with or without the guidance of an adult. As Ferrari suggests “The layout of the space is a fundamental factor in designing the activity and the game with the MIROR-Impro, we suggest setting up a quiet, silent and large enough space to allow movement.” (2015, 169–170).

Several experiences have been carried out, which suggest wider examples of how to use the MIROR platform in piano lessons with children (Benghi and Addessi, 2015; Nijs and Leman, 2015), as well with young children in classroom-based activities (Triantafyllaki et al., 2012; Ferrari and Addessi, 2014; Ferrari, 2015; Rowe et al., 2015), at home (Cardoso de Araújo, 2015), and for teachers’ education (Addessi, 2015).

Some scenarios are introduced below:

Scenario 1 – MIROR-Impro in the classroom and music school: Pupils improvise on the keyboard with the MIROR-Impro and learn to dialog based on repetition-variation, turn-taking, co-regulation. After several sessions with the MIROR-Impro, they will play with two keyboards, without the system, one pupil or a group of pupils for each keyboard, and will try to “dialog” only with the sounds, without using words. Pedagogical implication: learning to dialog and communicate with sound, learning musical phrasing, imitation and variation, co-regulation, turn-taking.

Scenario 2 – MIROR-Compo in the kindergarten and primary school: children create a story, draw the scenes of the story, and then compose the soundtrack of each scene using the MIROR-Compo. They can do it individually, in pairs, in small groups or as a class. The teacher acts as scaffolding and modeling, guides the children in the use of the MIROR-Compo, organizes the setting, the materials, and supports the children in expressing their musical ideas.

Scenario 3 – MIROR-Impro in the music classroom. *Teacher’s task assignment*: “explore with the system the following musical qualities: the duration of the sounds (long and short), the register (high and low pitch), the density (many sounds/few sounds).” The pupils play the keyboard along with MIROR-Impro, individually or in pairs. Time after time, they improvise dialogs with the system employing long or short sounds, high or low, dense or rare. The teacher observes and analyzes the musical dialogs that the children accomplish together with the system and supports their inventions. *Re-launch 1:* draw a graph that represents the sound dialog; *Re-launch 2*: explore other sound features: fast/slow, loud/soft, light/heavy, crescendo/diminuendo. Pedagogical implication: the task assignments of the teacher should be suitable for the musical level of the pupil and should involve more and more complex task assignments related to the musical, technical and performance goals of the curriculum.

Scenario 4 – MIROR-Body Gesture in the nursery, kindergarten and primary school, children with special needs, dance school: children, alone or in pairs, go into a special sensorized space, called the “planet of heavy and light things,” while the others wait outside. To make heavy or light movements, the children prepare themselves by rubbing a feather or a stone on different parts of their body, to become heavy or light. Then children start to walk, jump, skip, fall, etc., in a heavy or light way, listening and interacting with the sound produced by the MIROR-Body Gesture. Pedagogical implication: experiencing the heavy and light qualities of gestures and of sounds; improvising and composing the soundtrack of their own movements.

## Empirical Studies With Children and the Miror Platform

The educational effectiveness of the reflexive interaction paradigm has been demonstrated through the pioneering studies carried out since 2003 (Addessi and Pachet, 2005, 2006; Ferrari and Addessi, 2014) and then in the framework of the MIROR project (Addessi et al., 2015a, 2017a; Rowe et al., 2015). These studies, at the same time, also identified a number of critical issues of MIROR applications and compiled lists of requirements to improve these technologies in terms of their teaching functionalities.

### Creativity and Flow Experience With MIROR-Impro

The Flow state is described by Csikzsentmihalyi (1996) as the “optimal experience” lived by creative people while they are doing their favorite activities, and it is perceived by the subject as a balance between the goals she/he wants to achieve and the skills that the subject possess to achieve these objectives. The flow experience is characterized by the presence of high levels of intensity of several variables, which are: clear objectives, clear and immediate feedback, focused attention, control of the situation, pleasure, no worry of failure, changing of the perception of time, self-consciousness disappeared. The studies carried out by Custodero (2005) revealed that the flow theory could be an effective tool to approach children’s musical creativity.

We implemented an observational grid to study and measure the *flow experience* of children in a reflexive environment (Addessi et al., 2006, 2015a). The basic idea of our grid is that the observer does not register the flow state, but rather each variable and its intensity (from 1 to 3 levels of intensity). In accordance with Csikzsentmihalyi (1996), flow was considered present when all variables were registered by the observer at the highest level (level 3). Other combinations of the intensity levels of behaviors determined the state of arousal, control, anxiety, relaxation, worry, boredom and apathy. We adapted Csikszentmihalyi’s definition of each variable to the musical experience and proposed a detailed description of the musical behaviors of each variable, as follows:

• “*Focused attention* is an analytic behavior of great intensity, and is present when the child focuses on one or more particular elements. The child is not distracted by the environment. Some examples of behavior that characterize focused attention are: the child looks attentively at the keyboard and/or other elements of the equipment (loudspeakers, monitors, cables, etc.); the child observes, s/he is attentive and systematically explores some parts of the keyboard or other equipment; ….” (Addessi et al., 2015a, p. 134).
• Csikszentmihalyi defined *clear-cut feedback* as “internalizing the field’s criteria of judgment to the extent that individuals can give feedback to themselves, without having to wait to hear from experts” (1996, p. 114). In our observations we considered how the child analyses the feedback received from the system or the other child, for example: “the child becomes aware of the system’s response and s/he reacts by smiling or saying something; (…) changes her/his musical proposal according to the response received from the system”, etc. (*ibid.*).
• *Clear goals:* according to Csikszentmihalyi, “the creative process begins with the goal of solving a problem that is given to a person by someone else or is suggested by the state of the art in the domain (…). In flow we always know what has to be done” (Csikzsentmihalyi, 1996, p. 113). In fact, “children spontaneously create goals during the interaction with the system (…) the child clearly aims to explore the parts of the keyboard and the elements of the equipment; (…) different gestures to produce sounds: (…) the sounds of the keyboard and develop a musical idea” (*ibid.*).
• The variable *Control of situation* is characterized by the fact that “we are too involved to be concerned with failure, like a feeling of total control” (Csikzsentmihalyi, 1996, p. 112). Examples of *Control of situation*: “the child understands quickly that s/he can interrupt the system when s/he wants; self-assignment (…); deliberate gesture (…); the child explores and uses the equipment spontaneously, independently and with agility; (…) knows how to use/manage the rules of the interaction with the system (…) and with the partner” (*ibid.*).
• *Pleasure:* the “flow is an innately positive experience, it is known to produce intense feelings of enjoyment” (Csikszentmihalyi and Csikszentmihalyi, 1988, p. 35). Examples of behavior that characterize *pleasure*: “the child smiles and/or laughs, s/he is calm; (…) shows no displeasure; (…) repeats an action that s/he likes to do, for example, exploring a musical idea, playing and listening to the system, making a particular gesture, (…); (…) produces exclamations of pleasure, for example, ‘it answers me!’ or ‘it’s fantastic!” (*ibid.*).

We implemented the grid with the software Observer (by Noldus) and we planned to record second-by-second the *presence*, the *frequency*, the *duration*, and the *level of intensity* of each variable (level 1 = low intensity; level 2 = medium intensity; level 3 = high intensity). The flow is then measured by the software Observer that indicates the state of flow when all variables reach the highest levels of intensity (level 3).

Twenty-four children were involved in the experiments: 4 and 8-year-old children. They were asked over 3 sessions on 3 consecutive days to play a keyboard, with and without the MIROR-Impro, alone or with a friend. Furthermore, two different set-ups of the system were used: with group A the answer of the system was more “reflexive,” i.e., more similar to the child’s input (set-up Same), while with group B the system’s reply was less “reflexive,” that is less similar to the child’s input (set-up Very Different). All the sessions were video recorded and a video-analysis with the Flow grid was made in order to observe and measure the state of flow and the influence of the MIROR-Impro, the more or less reflexive answer, and the presence of the friend on the flow state of children during the sessions. Six observers were involved, all experts in children’s music education; two of them were members of the research team. The tool “Instructions for the observers” introduced the observers to the definition of each variable and to recording the variables by means of the software Observer. Several meetings were held to train the observers and to check the agreement among the observers. The reliability estimation and further statistical analyses were carried out with the same software Observer and SPSS. The results of the study confirmed the results of the first study, that is that the flow state increases when children play with the reflexive system, both alone and with a friend. Furthermore, the results also showed that the flow percentage is higher for group A, that is the group which played with the more reflexive set-up in all tasks. The difference between group A and group B (who played with the less reflexive reply) was significant (*p* = 0.004). In particular, the difference was significant in the 2 tasks with the system (*p* = 0.000; *p* = 0.001). Finally, the flow was higher in the tasks with the system, than in the tasks without the system, and the difference was significant, both in group A (*p* = 0.035) and B (*p* = 0.013).

#### Pedagogical Implications

The teacher can use reflexive systems and reflexive strategies (mirroring, turn-taking, and co-regulation) in order to enhance children’s *flow emotional state* within a creative experience. More precisely, to engage children in focused activity both when playing and listening (*focused attention*), with well controlled movements and the ability to manage the rules of interaction and the game with the partners (*control of situation*); to increase the activities started by the children (*self-assignment*), and their ability to play in a self-motivated way, without any external constraints (*clear goals*), to analyze the feedback produced by the partner (*clear-cut feedback*), to explore and play musical ideas, create fun games and play collaboratively (*excitement*).

### Meaningful Instrumental Learning and the Ability to Improvise

Improvisation is a very complex activity, as shown in the interdisciplinary approach of Berkowitz’s (2010). Improvisation can be considered an important support for the development of creative thinking, because it motivates children to use their imagination, self-regulation and intrinsic motivation (Custodero, 2005; Tafuri, 2006). We carried out an experimental study to investigate whether reflexive interaction with the MIROR-Impro influences children’s ability to improvise (Addessi et al., 2017a). Forty-seven children aged 7 and 8 years participated in the study, divided into three sample groups: control group, experimental groups no. 1 and no. 2. With the aim to verify if the reflexive interaction is *necessary* and/or *sufficient* to improve children’s abilities to improvise, we adopted an experimental design based on the convergence procedure (Campbell and Heller, 1979; Fiske, 1992). The independent variables were represented by three different conditions: (1) to play only the keyboard, (2) the keyboard with the MIROR-Impro using a not-reflexive reply, and (3) the keyboard with the MIROR-Impro using a reflexive reply. Each child carried out 5 weekly individual sessions. Each session lasted 12 min. The control group played according to all three independent variables; experimental group 1 played according to independent variables 1 and 2, that is only the keyboard and the keyboard with the MIROR-Impro with not-reflexive reply; finally, experimental group 2 played the keyboard according to independent variable 3, that is only with the reflexive system. One week after the training period ended, the children were asked to improvise a musical piece on the keyboard alone (Solo task), and in pairs with a friend (Duet task). In the Duet task, each child played a keyboard and they were asked to dialog only with the sounds, without words.

Three independent judges assessed the Solo and the Duet tasks by means of the TAI-Test of Ability to Improvise rating scale implemented by McPherson (1993, 2005). The TAI is a tool to evaluate musical improvisations by listening to them, based on four evaluative criteria: *Musical organization*, *Instrumental Fluency*, *Musical Quality, and Creativity*. For the Duet task the judges also received 3 new criteria focused on the interaction: the *Quality of Musical Dialog*, that is the “ability to dialog and interact with the partner by using the sounds: paying attention to the musical proposal (listening), the ability to reply in a way correlated to the friend’s musical proposal (e.g., by repetition, variation, contrast, etc.), the presence of symmetries, co-regulation, sharing and co-production of musical ideas, the ability to show a global intentionality to dialog with the friend” (Addessi et al., 2017a, p. 10); the *Reflexive Interaction*, that is the “ability to interact using repetition and variation, turn-taking, and co-regulation” (*ibid.*); the *Attention span*, that is the “the subjects’ tendency to persist in their contact with the activities, in this case the musical dialog with the other child, irrespective of any underlying aim” (*ibid.*). In the original article, for estimating the reliability of the computed mean value across the judges, we used Cronbach’s alpha (CA). The resulting CA indicated a high reliability (solo task, 0.93–0.96; duet task 0.94–0.99). The judges’ agreement in terms of the individual variation was estimated by the mean Pearson’s correlation *r* between all pairs of judges. The resulting correlations indicated a good agreement (solo task, 0.83–88; duet task 0.84–0.97). Subsequently, a more general measure for estimating the reliability was calculated, i.e., Krippendorff’s alpha (Krippendorff, 2004). The resulting alpha indicated that the ratings were reliable (solo task, 0.81–0.88; duet task, 0.83–0.96).

The experimental group 2, which trained only with the MIROR-Impro, reached the highest average results of all criteria, both when the children improvise alone and in a duet. In particular, in the Duet task the difference between the total score of ability to improvise of the experimental group 2 (4.34) and experimental group 1 (3.13), which did not use the MIROR-Impro, was statistically significant (*p* = 0.046). These results support the hypothesis that the reflexive interaction with the MIROR-Impro could be sufficient to increase the improvisational skills, and necessary when the children improvise in duets. In the Duet task, the correlation between reflexive interaction and the other criteria, including creativity, was high and statistically significant (*p* < 0.01), which could indicate that practice with the reflexive system “teaches” children the mechanisms of reflexive musical interaction (turn-taking, co-regulating their behavior with the partner, imitating, being imitated, repeating and varying) and then they are able to use these reflexive behaviors also when they interact with a human partner (see Figure 7). The results also show that the attention spans of experimental group 2 (children who trained only with MIROR-Impro: 585 s) are higher than the other two groups of children (control group: 428 s; experimental group 2: 515 s).

**FIGURE 6:**
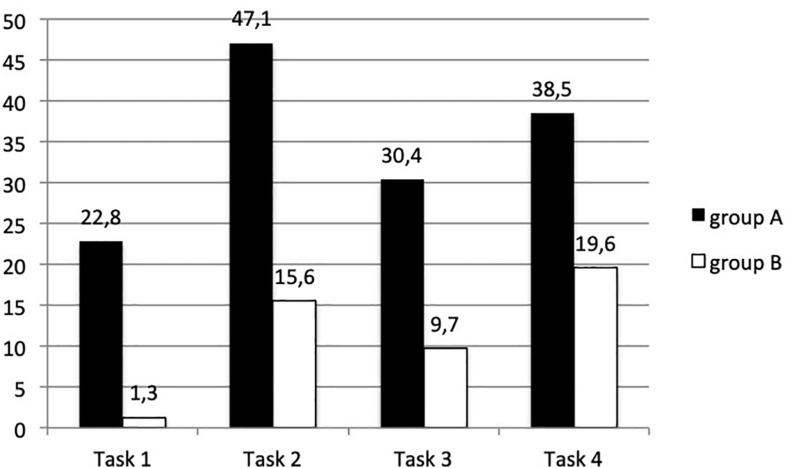
Percentages of flow with group A and group B in each task. In the tasks with the system (T2 and T4) group A used the set-up Same and group B used the set-up Very Different (Addessi et al., 2015a).

**FIGURE 7:**
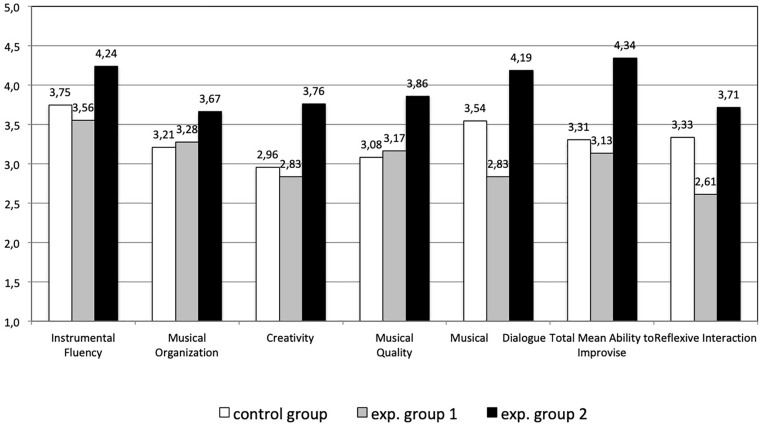
Duet task: scores for each evaluative criterion, total score of ability to improvise, and score of reflexive interaction (means) (Addessi et al., 2017a).

#### Pedagogical Implications

Reflexive technologies can support a music improvisation program by means of individual and collective “deliberate practice” (Ericsson, 1997; McPherson, 2005). The teacher can use reflexive interaction to enhance children’s creativity, that is: (1) Musical flexibility: the child’s ability to generate differing musical ideas; (2) Musical originality: the child’s ability to provide a musically unique or unusual response (McPherson, 2005). Furthermore, reflexive systems can enhance the quality of children’s musical dialog, their ability to musically interact with the partner, by paying attention to the musical proposal (listening), to co-regulate and share musical ideas, using repetition and variation, and turn-taking.

### Children’s Movement Creativity in a Reflexive Embodied Environment

In recent years, several studies proved that cognitive processes can be influenced by body states, both real and imaginary (Barsalou, 2008). The general hypothesis, at the base of this vision belonging to the field of embodied cognition, is that cognitive processes are closely linked to bodily states, or that knowledge is closely linked to the physical context (see Varela et al., 1993). The importance attributed to the relationship between action and perception has led to a particular attention to the body in its involvement with the musical experience. In the field of creative technology, some experiments have been carried out with children who interact with a machine through visual feedback, body movements, and listening (Friberg and Kallblad, 2011; Nijs et al., 2012; Frid et al., 2016; Sano, 2018). These studies show that motion capture tools have made clear progress in the quantitative analysis of movement and gesture. Nevertheless, the measurement of motor “creativity” still remains an open challenge.

In the framework of the MIROR project, we carried out several experimental studies aimed at investigating whether interaction in reflective musical environments can improve creative processes and children’s ability to improvise with movement. We noticed, in fact, that the reflexive response of interactive systems generates interesting motor reactions in children: several creative gestures to produce the sounds or dancing while listening to the system’s response. These observations raise many questions: what is the children’s bodily perception when they hear the reflexive response of the system? Which and what sort of movements does the child imagine? How do the system’s responses stimulate the child to create a gesture connected to the sound? We can hypothesize that reflexive interaction can stimulate a body *resonance* mechanism in the child, as described by Rizzolatti et al. (2002), since this mechanism is rooted in the motor areas of the brain. When children dance while listening to the reflexive answers, they act as if they were “incorporated” mirrors of the system’s musical response, so that the interaction of the child with the machine also sees a channel of body communication activated.

We first listed several requirements of the reflexive “embodied” interaction for the implementation of the MIROR-Body Gesture. The second level of investigation concerned the relation between the child’s movements and the sound produced by the system. According to Godøy and Leman (2010, p. 6), the “analysis of sound, in particular the movements in sound, can therefore be used as a starting point in identifying sound-related musical gesture.” In the case of a reflexive system, this means that the related sound and gesture should give children the perception that the sound is a sort of virtual copy of her/his gestures. Aiming to implement a reflexive sound-related musical gesture, the UNIBO team created a grid of correlation between Laban movement parameters (Laban, 1950/1980) and musical features (Baroni, 2004). The particular interest of this grid is that the musical qualities were obtained by observing children making sounds, and by interviewing them (Addessi et al., 2013b, 2015b).

In order to measure the improvement of the quality of children’s movements, we used the TCAM test (Torrance, 1981) and implemented an original grid based on the Laban Movement Analysis (LMA) (Laban, 1980). The TCAM was conceived to measure some types of creative skills in children between the ages of three and eight, namely originality (assessed according to the criterion of statistical frequency), fluidity (the number of different and appropriate responses), and imagination (how the person is able to imagine and adopt the various roles proposed). The LMA, which was originally created to describe, visualize, interpret and document human movement, in our case was used with a more specific application in the field of dance and movement education. This approach has been used with excellent results in the field of musical studies (e.g., Broughton and Davidson, 2016). In our experiment we observed four factors of the category *Effort*, which are: Space (direct or indirect), Time (sustained or sudden), Weight (light or heavy), and Flow (free or bound).

An experimental study was carried out with the MIROR-Impro to investigate if reflexive interaction can enhance the creativity of children’s movements (Addessi et al., 2017b). It was realized in Italy in two classes of the first cycle of a public primary school, with 47 children aged 7 and 8, divided into two groups: control group (24 children) and experimental group (23 children). Both groups participated in 4 lessons, one each week. In each lesson all children were asked to improvise different activities with the body while they were listening to a child playing a keyboard. However, the child of the control group only played the keyboard, while the child of the experimental group played the keyboard with the MIROR-Impro. So the children of the experimental group improvised different activities with the body while listening both to the child and the reflexive responses of the system. An example of an activity carried out:

On the moon. We propose to the children to pretend to be in a science fiction film set on the Moon: “Pretend to be animals, aliens and rocks of a lunar landscape.” The child-musician therefore has the task of playing the soundtrack of a science fiction film. To the rest of the class we propose alternatively: “move like flying animals during the sound proposal of your musician-partner, and move like creeping animals during the computer response”; “Move like rocks that roll during the proposal of the music-companion and freeze in a position during the computer response.” The children took turns in the role of musician. The same activities carried out with the experimental group were carried out with the control group, but the child who played did not have the reflexive response of the system and the children who danced responded with movement only following the sound proposed by the musician-partner.

We used a modified version of Activity 2 “Can you move like that?” of the TCAM test by Torrance to measure the children’s motor creativity before and after the activities. This test is suitable for measuring the child’s ability to imagine and take on different roles by moving like animals or objects, and which then evaluates the imaginative capacity. For example: “Can you move like a tree in the wind? Imagine you are a tree and a wind is blowing very hard. Show me how you would move by moving forward toward the camera” (*ibid.*).

The activities of pre-test and post-test were analyzed as reported in the administration, scoring, and norms manual of the TCAM Torrance test. Each task was rated with a score from 1 to 5, on the basis of the quality, adequacy, and elaboration of each movement. Two judges, i.e., the dance teacher and the researcher who carried out the experimental protocol, were required to independently watch the videos of pre and post-test activities and to evaluate the children’s performance using a 5-point scale. The control group and the experimental group showed no difference in the results of the TCAM test performed before the activities (*M* = 26.1 vs. 26.8, *p* = 0.62), while a significant difference emerged between the two groups after the activities. In particular, and in line with our hypothesis, there was an evident increase in the creativity scores of the experimental group, which had performed activities with the MIROR-Impro reflexive system, compared to the control group (control group *M* = 28.8 vs. experimental group *M* = 31.8, *p* < 0.05). These results support our hypothesis that reflexive interaction, thanks to its mirroring mechanisms, turn alternation, regulation and co-regulation, positively influences the development of motor creativity in children.

The qualitative analysis using the LMA highlighted some qualities of movement and use of Space. In particular, in the post-test phase, we observed that the children of the experimental group showed, compared to the control group: a wider kinesphere (the sphere that limits or delimits the personal space of the movement), with a consequent exploration of gestures, both of the arms and of the legs, more extended and defined – for example, during the post-test, in task 1 “Can you move like a tree in the wind?”, it was noted that, to simulate the movement of the tree crowns, the amplitude of the gesture of the arms was greater in the experimental group than in the control group; a safer use of the general space; greater use of individual parts of the body (arms, shoulders, head, and feet).

#### Pedagogical Implications

The usefulness of the reflexivity paradigm is that the children remain with thought-movement on the same activity while elaborating variations. This allows the teacher to organize activities that support children in experimenting with various body responses to musical proposals and vice versa, placing greater attention on the relationship between elements of music (sound, melody, rhythm, etc.) and elements of movement (body, space, relationships, time, etc.). It is important that children express themselves without going through verbal language, but taking on “other” languages.

### MIROR-Compo: A Scaffolding for Creating the Musical Shape

We collected a database of children’s composition and of musical phrases chosen by the children, as *Initial Statement*, *Continuation*, *Variation*, *Answer*, and *Conclusion*. The compositions and the musical phrases were analyzed in order to understand how children used the *actions* of MIROR-Compo. It was possible to observe constants in the way each type of *action* is linked to the previous or next sentence. For example the Variations often show a change of register with respect to the last note of the previous sentence; all the Conclusions show an ascending melodic profile, repeated notes, and are of greater duration than the previous sentences. Therefore, the function of a musical phrase is not in the musical content itself, but in the way in which each musical phrase is linked to the others. This shows us how the compositional process stimulated by the system does not so much concern the creation of musical phrases, but rather the creation of a form, that is, the way of combining and putting together the musical phrases (literally, com-pose).

This is very interesting because it supports the idea that the MIROR-Compo can act as a kind of “musical *scaffolding*,” which allows children to invent different musical compositions, depending on their sense of form. The concept of “scaffolding” (Vygotsky, 1962; Bruner, 1983) offers the metaphor of this experience: the system provides subsequent levels of temporary support (in this case, the musical phrases) that help children to achieve other levels of understanding and skills (the overall form of the composition), which would not be possible without a guide. As in “scaffolding,” the support strategies implemented by the system are gradually removed by the system itself, when the child no longer needs them, and the responsibility to create the musical form gradually becomes the task of the child. Furthermore, the MIROR-Compo can also be used as a bridge to fill the so-called “learning gaps,” that is, the difference between what the child has already learned and knows because of her/his acculturation (e.g., their understanding of a musical phrase with a beginning, a development and a conclusion), and what s/he expects to be able to do with it, such as composing a sequence that satisfies her/his sense of form and musical narrative.

### Reflexive Technology in Inclusive Contexts

Several empirical experiments and practices have been implemented in the framework of the MIROR project, with meaningful results using reflexive interaction with adults with autistic syndrome and with children in a music-therapy setting (Anagnostopoulou et al., 2012; Bonfiglioli, 2015). More recently, further studies are ongoing in order to test the MIROR application in inclusive educational contexts (Ferrari and Addessi, 2016, 2019), with children with impaired hearing (Gurioli et al., 2019), and in dance schools with children in wheelchairs (Bertocchi, 2017).

These studies showed that reflexive interaction can be a “transversal” device for creativity, music education and music therapy, and can enhance expressive and creative behavior in situations of disability and/or in which it is important to promote inclusion. The flow experience generated by the interaction with MIROR applications favors states of creativity and well-being, suggesting an effective therapeutic and rehabilitative potential. Reflexive interaction stimulates specific brain areas of resonance and activates interactive processes that deeply involve the person. Nadel (2002) points out that imitation and recognition of imitation are fundamental for understanding the autism syndrome. According to Rizzolatti et al. (2002), autism may have a neurobiological basis in the malfunction of mirror neurons. The reflexive interactive musical systems can therefore be placed at the crossroads between music education and music therapy, where the music therapist’s task is to set, through listening, the conditions to promote creativity and social processes (Bunt, 2012). In particular, they are adaptive and intuitive systems, analogous to the extemporaneous character of music therapy improvisation. They are based on the co-regulation of a communicative process defined as “a continuous disclosure of the individual action that is susceptible to introducing new actions from the constantly changing actions of the partner” (Fogel, 2000). Further constitutive characteristics of reflexive interactive musical systems useful for inclusive education are the *priority given to child/ren and to their musical style(s) and identity(ies), the child-centered learning approach*, a *tool for the children to express themselves*, their emotions and symbolic imaginations, by means of the body and the music, the *interaction based only on sound feedback* (no need for music notation or the computer screen), the *collaborative learning*, the *direct peer learning*, the *self-organization* of groups.

## Conclusion

In this article, an overview of the pedagogical paradigm of reflexive interaction was presented and its application in the field of technology-enhanced learning, that is the MIROR platform. This paradigm has proven effective in various area of children’s creativity and instrumental learning, suitable for different ages and in different areas of musical experience, from exploration to improvisation, composition and motor creativity, in individual or collaborative activities, with the teacher’s guidance or in deliberate practice.

With this work we have proposed an original technology for children’s embodied music and creativity. The mechanism of repetition-variation, which is at the heart of reflexive interaction, gives rise to a process of co-regulation between children and the machine, where the center of attention is not the final product but the subject engaged in the interaction. That is, the machine does not require the child to achieve pre-determined objectives, but it co-constructs these objectives with the child, motivating the child to develop original musical and motor ideas. This creates a novel kind of child-machine interaction that was proved capable of having a particular impact on teaching and learning processes.

This overview has made it possible to compare all the stages of the project and therefore to offer a complete and more significant vision of the reflective interaction paradigm and its applications in the pedagogical field, starting from the first intuitions that emerged during the pilot study observations, to the construction of the theoretical framework, which has been enriched over time with paradigms of the Western scientific tradition (from Flow theory, to Vygotskian constructionism, mirror neurons, embodied cognition and enactive approaches), to the qualitative and quantitative results of controlled experimental protocols and empirical investigations conducted with children from 2 to 10 years, in different scenarios: nursery, kindergarten, compulsory school, music school, dance school, inclusive and music-therapeutic contexts.

The basic hypothesis, namely that the reflexive interaction enhances musical and motor creativity, arose from the observations collected in the pilot studies with children. The subsequent MIROR project led to the implementation of the MIROR platform, with the extension of the reflexive interaction paradigm from musical improvisation to composition and embodied cognition. The project also led to the conceptualization of the pedagogical framework of reflexive interaction, and to the rigorous experimentation of different scenarios, with different ages and groups of participants, with greater attention to the role of adults and teachers. This article presented the main results obtained during the project.

The use of qualitative and quantitative approaches, according to the mixed methods (Teddlie and Tashakkori, 2009), was found to be effective and allowed to support the qualitative observations conducted in the field with quantitative data, thus allowing to fruitfully connect the children’s and teachers’ daily experiences with controlled experimental protocols and scientific evidence, which is still a problem in the field of pedagogical research. The interdisciplinary and mixed methods approaches led researchers to implement an original, highly promising research methodology in the field of psycho-pedagogical sciences.

The spiral approach, which saw the collaboration between music education and music technology experts, demonstrated that “an interdisciplinary approach and collaboration between hard and soft sciences is feasible and that the development of technology in a context of psychology -pedagogy can be mutually fruitful” (Final report EU). As written by the reviewers of the European commission “The technology provided new stimulations for thinking out of the box about new pedagogy and child-machine interactions.”

From these points of view, the project has achieved its objectives. However, there are further issues on which it is necessary to continue improving, such as the user’s interfaces, the application of MIROR-Body Gesture, the wider application in different scenarios of music education.

From the theoretical-experimental point of view, the results open the door to new hypotheses about the interconnection between the reflexive interaction paradigm and the theories of mirror neurons and embodied cognition: in this direction a new application is on the road, called MIROR-MultiModal, which combines sound, movement and colors, and which should allow to realize the idea of a “reflexive” space in which children can create music and images with the movement of their body. The reflexivity paradigm has also been extended to studies of vocal interactions between children and adults. It has been observed that the elements that characterize the reflexive interaction, i.e., mirroring, turn-taking, co-regulation, are fundamental in adult-child vocal interaction in early childhood and represent one of the main mechanisms of a child’s vocal activism (Addessi, 2020).

The research techniques developed during the project were used in subsequent projects such as in the LINK project (Learning In a New Key, Erasmus Plus, EU). The flow grid implemented in the MIROR project was used to observe the impact of training courses, carried out by non-music specialist teachers together with music therapists on some basic principles of music and art therapy, on the class-room activities with children of greater cognitive and social vulnerability (Tarr and Addessi, 2017).

Since the end of the MIROR project, many experiments have been conducted and continue to be conducted in many schools and research centers, in particular in inclusive contexts (Bertocchi, 2017; Ferrari and Addessi, 2019; Gurioli et al., 2019; Figueiredo et al., in press), with other instruments, such as percussion (Pscheidt et al., 2019), and in piano teaching (Hamond et al., 2019). Many doctoral and postdoctoral students are testing the platform in different educational and teaching settings, also expanding the theoretical discussion, such as the comparison between reflexive interaction and Cross’s concept of empathetic creativity (Cross et al., 2012; see Pscheidt et al., 2019).

These recent experiences support the idea that the reflexive paradigm and the MIROR applications represent a powerful and adaptive device for children’s instrumental creativity and learning, and contribute to improving these systems, in order to discover new scenarios and new theoretical and technological issues and hypotheses.

## Data Availability Statement

The datasets generated for this study are available on request to the corresponding author.

## Ethics Statement

The studies involving human participants were reviewed and approved by University of Bologna. Written informed consent to participate in this study was provided by the participants’ legal guardian/next of kin. Written informed consent was obtained from the individual(s), and minor(s)’ legal guardian/next of kin, for the publication of any potentially identifiable images or data included in this article.

## Author Contributions

The author confirms being the sole contributor of this work and has approved it for publication.

## Conflict of Interest

The authors declare that the research was conducted in the absence of any commercial or financial relationships that could be construed as a potential conflict of interest.
